# Spatial-Variant SAR Range Cell Migration Correction Using Subaperture Strategy

**DOI:** 10.3390/s21072444

**Published:** 2021-04-01

**Authors:** Liping Hu, Guanyong Wang, Lin Hou

**Affiliations:** 1 Science and Technology on Electromagnetic Scattering Laboratory, Beijing Institute of Environmental Features, Beijing 100854, China; fox_plh@163.com; 2 Beijing Institute of Radio Measurement, Beijing 100854, China; 3 Beijing Aerohydrodynamic Frontier Research Center, Beijing 100074, China; houlin55@126.com

**Keywords:** synthetic aperture radar (SAR), highly squinted, subaperture, range cell migration correction (RCMC)

## Abstract

The coupling between range and azimuth dimensions is the main obstacle for highly squinted synthetic aperture radar (SAR) data focusing. Range walk correction (RWC) processing is effective to remove the linear coupling term, but the residual high order range cell migration (RCM) parts are spatial-variant in both range and azimuth dimensions. In this paper, we propose a precise spatial-variant range cell migration correction (RCMC) method with subaperture processing. The method contains two stages. Firstly, the main component of range-variant RCM is corrected in the coarse RCMC stage. Secondly, data are derived into azimuth subapertures (SAs), an SA-image-domain RCMC is developed by interp correction, where the SA image is obtained using a modified spectrum analysis (SPECAN) algorithm by establishing the relationship between Doppler frequency and residual spatial-variant RCM. In the proposed algorithm, precise compensation of space-variant RCM is implemented by SA processing, which is designed for a better practicality in real-time processing system. Simulated and real measured data experiments are designed to validate the effectiveness of the proposed approach for highly squinted SAR imaging.

## 1. Introduction

The highly squinted operating model [1,2] is widely used in the practical application of airborne synthetic aperture radar (SAR) [3], which has the advantages of higher mobility and flexibility to observe the areas of interest. However, the signal coupling of the range and azimuth in the highly squinted mode is more serious than that in the broadside mode, which also brings more problems for signal processing. Especially for unmanned aerial vehicle (UAV) [4,5], platforms and other maneuverable platforms [6,7,8], a high-precision and robust imaging algorithm in the highly squinted mode is important in actual applications. For highly squinted SAR imaging, the most accurate method is based on time domain processing, such as backprojection (BP) and its extended fast imaging algorithms [9,10,11]. Although this kind of method implements the highly squinted imaging accurately, it is difficult to use in hardware applications due to the large computational burden. Another kind of method is based on frequency domain decoupling. Omega-k algorithm (wkA) [12,13] decouples the range and azimuth signal of highly squinted SAR in a two-dimensional wavenumber domain. However, high pulse repetition frequency (PRF) is required in actual applications, which will bring difficulties in terms of data operation and storage. In addition, it is more difficult to combine wkA with a motion compensation algorithm [14].

In real measured data processing, it is effective to remove the linear component of highly squinted data by range walk correction (RWC), which significantly simplifies the decoupling process. However, RWC leads to an unexpected result that both range cell migration (RCM) and azimuth matching function become azimuth-variant [15], that is, they change with the position of target in the azimuth direction, which will introduce non-negligible errors for wide-beam and high-resolution imaging. A typical method for solving this problem is azimuth nonlinear chirp scaling (ANLCS) [16]. This method uses the nonlinear chirp perturbation function to balance the azimuth frequency modulation (FM). On the basis above, a series of modified methods [17,18] extend the application scope and improve the accuracy to a certain extent, but these methods cannot compensate for the residual azimuth-variant RCM. In terms of this issue, an azimuth-dependent quadratic RCMC method is proposed in [19] by azimuth space variation filtering. A method is proposed in [20] to compensate the residual RCM envelope in the fourth-order approximation. The second order compensation for residual azimuth-variant RCM by extending ANLCS is realized in [21]. The compensation precision of ANLCS is then improved to a cubic phase term in [22]. Authors in [23] accurately calculated the expression of residual azimuth-variant RCM, and realized the accurate compensation of RCM by image post-filtering. The methods mentioned above are all implemented with full aperture processing, but in practical application, due to the influence of platform mobility, full aperture processing is not easy to achieve with accurate motion compensation. Hence, SA processing makes it more convenient and adjustable to process on a real-time processing system, not only because of the flexibility to combine with the motion compensation method, but also because SA processing can be implemented by parallel operation on the hardware.

Time-varying step transform [24,25,26] is one of the classic subaperture (SA) processing methods. In this method, the azimuth-variant FM is balanced by multiplying each SA with the corresponding compensation function. Based on [27], SA processing is introduced to speed up the conventional ANLCS. However, the azimuth-variant RCM is not considered in the above methods. Based on the principle that azimuth times corresponding to the same Doppler frequency are different for targets in the same range cell, an effective SA azimuth-variant RCM compensation method is proposed in [28]. However, the selection of the length of SA should be long enough to reduce the spectrum leakage, which limits the compensation accuracy of azimuth-variant RCM.

Therefore, based on the analysis above, an SA processing-based method is investigated in this paper, which implements the accurate azimuth-variant RCM compensation for highly squinted SAR. The proposed method contains two stages. Firstly, after RWC processing, the main component of range-variant RCM is corrected in the coarse RCMC stage by range chirp scaling algorithm (CSA). The analytic expression of residual RCM after RWC and coarse RCMC with CSA is derived by using the principle of stationary phase (POSP) [29]. Secondly, data are derived into azimuth SAs. Based on the analytic expression, an SA-image-domain RCMC is developed by interp correction, where the SA image is obtained by using a modified spectrum analysis (SPECAN) algorithm [29]. The SA-image-domain RCMC is named as the fine RCMC, and the key step is to establish a precise mapping relationship between the SPECAN image coordinates and the real target coordinates. In the proposed approach, the method of series reversion (MSR) [30] is applied to develop this mapping relationship. After the fine RCMC, accurate azimuth compression is implemented by the azimuth equalization of Doppler rates with the ANLCS. The original idea of this paper comes from our former research in [31], we further improve the algorithm that range CSA is applied to compensate the main component of range-variant RCM. The residual spatial-variant RCM introduced by RWC changes slowly in range direction so that it could be corrected by a rang scaling process in the SA image domain, we analyze the feasibility of the method in this paper. The proposed method does not require range blocking, so the processing complexity is further reduced.

The paper is organized as follows: Section 2 gives the flowchart of the method. Section 3 gives the signal and geometry model of the highly squinted SAR and introduces range cell migration analysis for highly squinted SAR. Section 4 presents the principle of the fine RCMC and its detailed implementation in highly squinted mode. In Section 5, we present the experimental results with both simulated data and real measured data. Conclusions are given in Section 6.

## 2. Method

**Figure 1:**
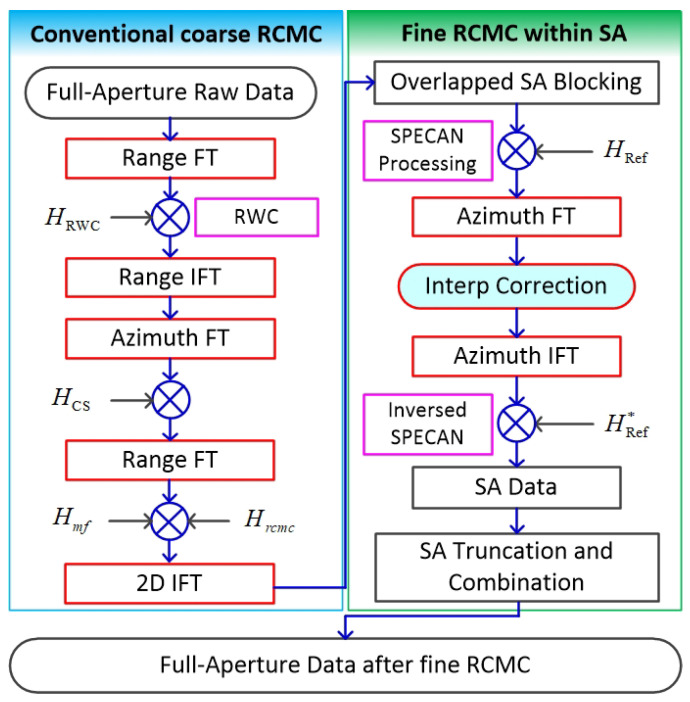
Flowchart of the proposed range cell migration correction (RCMC) method for highly squinted synthetic aperture radar (SAR).

In this section, we firstly present a flowchart of the proposed RCMC method and detailed step-by-step instructions. A flowchart of the proposed RCMC algorithm is given in Figure 1. It contains two main stages, which are conventional coarse RCMC and fine RCMC by SA process. Some main steps of the flowchart are illustrated as follows.

(a) RWC processing. Squint angle minimization is implemented in this step, so that the linear coupling term of highly squinted SAR data is eliminated by multiplying RWC function $H_{\mathrm{RWC}}$.

(b) Chirp scaling. This step is performed in the range time and azimuth wavenumber domain, in which a chirp scaling function $H_{\mathrm{CS}}$ is constructed to perform scalar transform to the data.

(c) Range matched filtering. This step contains the fine second-order coupling terms correction, so that the range direction achieves complete compression. The matched filtering function is shown by $H_{\mathrm{mf}}$.

(d) Coarse RCMC. The bulk RCM is corrected with respect to the reference point in this step, which is processed by multiplying RCMC function $H_{\mathrm{CS}}$ in a two-dimensional wavenumber domain.

(e) Azimuth SA blocking. The range blocking data set is divided into several SAs in azimuth, then we have a number of SAs.

(f) SPECAN processing. This step involves a phase function $H_{\mathrm{Ref}}$ multiplication and azimuth Fourier transform (FT) processing. Then, a coarse resolution image is obtained in the azimuth SPECAN image domain by azimuth FT processing.

(g) Fine RCMC. In the azimuth SPECAN image domain, the azimuth-variant residual RCM is easy to compensate by integrally shifting the image in the range direction according to the azimuth coordinate. This step is rapidly implemented by interp correction processing. As is shown in the flowchart, interp correction is given in the SPECAN image domain, which is the core step to achieve fine RCMC. The key to fine RCMC is to accurately calculate the displacement error of each SPECAN image point caused by the large squint, so we can perform the deformation correction to the SPECAN image based on the accurate displacement error expression.

(h) Inversed SPECAN processing. Similar to SPECAN processing, this step involves an azimuth inverse Fourier transform (IFT) processing and a phase function $H_{\mathrm{Ref}}^{*}$ multiplication. The residual range and azimuth-variant RCM is fully compensated in each SA.

(i) SA truncation and combination. By merging all the SAs, the full-aperture data after fine RCMC is obtained.

In the following sections, we will explain the principle of this method in detail, and all the symbols in the flowchart will be explained.

## 3. Range Cell Migration Analysis for Highly Squinted SAR

### 3.1. Geometric Model

**Figure 2:**
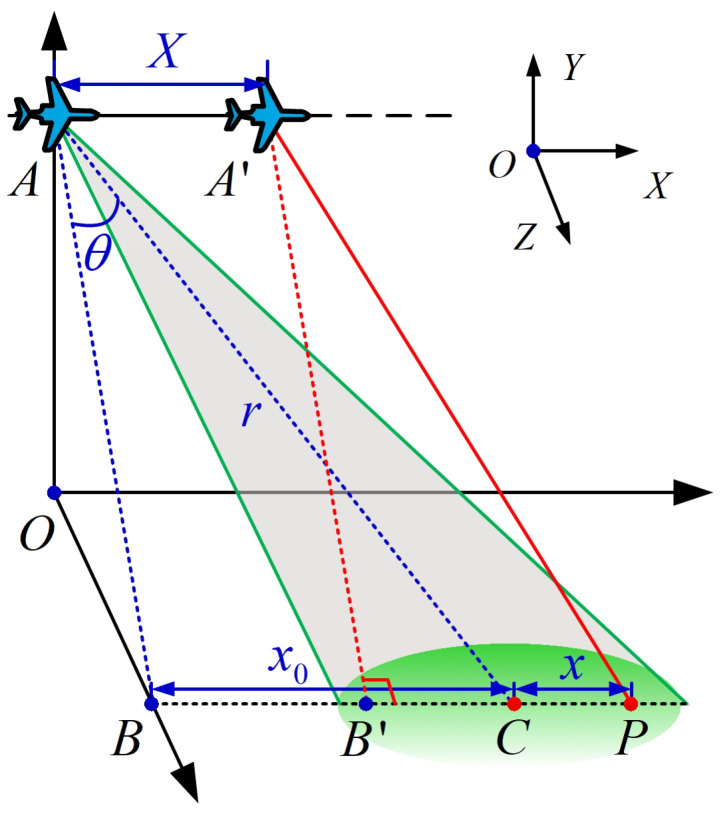
Highly squinted SAR geometric model.

The geometric model of highly squinted SAR imaging is shown in Figure 2, which is defined in a three-dimensional Cartesian coordinate system named O−XYZ. In the ideal case, the platform moves along the *X* axis at a constant velocity *v* and generates a synthetic aperture with *L*. The squint angle is defined as θ during the system operation. At initial time $t_{0}$, the slant distance from the sensor at *A* to the scene center *C* is *r*. The platform moves to $A^{\prime }$ at time $t_{1}$, and the traveled distance is *X*, so we have $X=(t_{1}-t_{0})v$. Symbol P stands for a target point located on the scene center line, and *x* stands for the distance between *P* and *C*.

During the operation time of the radar system, the antenna transmits the linear frequency modulation (LFM) signal and receives the echo reflected from the ground. Ignoring the antenna pattern attenuation, the echo of P received and down-conversed by the radar is given by [4]

(1) $$\begin{array}{c} s\left(t_{r},X\right)=\varepsilon_{p}\cdot \mathrm{rect}\left(\frac{t_{r}-\Delta t}{T_{p}}\right)\cdot \mathrm{rect}\left(\frac{X-x_{0}-x}{L}\right)\\ \;\;\;\;\;\;\;\;\;\;\;\;\;\;\cdot \exp\left[j2\pi \left(-f_{c}\Delta t+\frac{1}{2}\gamma {\left(t_{r}-\Delta t\right)}^{2}\right)\right] \end{array}\;$$

where, $rect\left(\cdot \right)$ represents the rectangular window function. $\varepsilon_{p}$ corresponds to the complex-valued scattering amplitude of the point target. $t_{r}$ denotes the range fast-time, and $t_{m}$ is the azimuth slow-time. $T_{p}$ denotes pulse duration width, *L* denotes the synthetic aperture length, fc represents the carrier frequency and γ is the chirp rate of the LFM signal. Δt is the round-trip delay from radar antenna phase center to *P*, which can be expressed as Δt=RpRp2c2c. *c* is the speed of light in the atmosphere. According to the geometry in Figure 1, the instantaneous range $R_{p}$ from *P* to radar antenna phase center is given by [23]

(2) $$R_{p}\left(X,x,r\right)=\sqrt{{\left(r\cos\theta \right)}^{2}+{\left(X-x-r\sin\theta \right)}^{2}}$$

We can polynomially expand (2) and find that there is a serious coupling in the data at a large squint angle θ. Data decoupling is then performed to make the data independent in range and azimuth directions. The general decoupling method is implemented in two steps, which are RWC and RCMC. We would theoretically analyze the problem of conventional decoupling process in the following subsections.

### 3.2. Range Walk Correction

In this subsection, we derive the signal expression after RWC processing in detail. The RWC is firstly applied to remove the linear component of data. Apply range FT to (2), and we have

(3) $$\begin{array}{c} S\left(\Delta K_{r},X\right)=\int s\left(t_{r},X\right)\cdot \exp\left(-j\Delta K_{r}\hat{r}\right)d\hat{r}\\ =rect\left(\frac{\Delta K_{r}c}{4\pi \gamma T_{p}}\right)\cdot \mathrm{rect}\left(\frac{X-x_{0}-x}{L}\right)\cdot \exp\left[-j\frac{\Delta K_{r}^{2}c^{2}}{16\pi \gamma }\right]\\ \;\;\cdot \exp\left[-jK_{r}\cdot \sqrt{{\left(r\cos\theta \right)}^{2}+{\left(X-x-r\sin\theta \right)}^{2}}\right] \end{array}$$

where, $K_{r}$ stands for the range wavenumber spectrum with $K_{r}=\Delta K_{r}+K_{rc}$, $\Delta K_{r}$ is the normalized range wavenumber spectrum with ΔKr∈−2παTp2παTpcc,−2παTp2παTpcc. r^=trctrc22 represents the range variable. To minimize the linear coupling, RWC function is defined as

(4) $$H_{RWC}\left(\Delta K_{r},X\right)=\exp\left(-jK_{r}X\sin\theta \right)$$

Multiplying (3) with (4), the signal expression is given by

(5) $$\begin{array}{c} S\left(\Delta K_{r},X\right)=\mathrm{rect}\left(\frac{\Delta K_{r}c}{4\pi \gamma T_{p}}\right)\cdot \mathrm{rect}\left(\frac{X-x_{0}-x}{L}\right)\cdot \exp\left[-j\frac{\Delta K_{r}^{2}c^{2}}{16\pi \gamma }\right]\\ \;\;\;\;\;\;\;\;\;\;\;\;\;\;\;\;\;\cdot \exp\left[-jK_{r}\left(\sqrt{{\left(r\cos\theta \right)}^{2}+{\left(X-x-r\sin\theta \right)}^{2}}+X\sin\theta \right)\right] \end{array}$$

To obtain the 2D wavenumber spectrum, we apply azimuth FT to (5), shown as follows

(6) $$S\left(\Delta K_{r},K_{x}\right)\;=\int S\left(\Delta K_{r},X\right)\exp\left(-jK_{x}X\right)dX$$

where, $K_{x}$ represents the azimuth wavenumber spectrum. According to the previous analysis in [23], we ignore the envelope expression, the 2D wavenumber domain spectrum of RWC processed signal is approximately given by

(7) $$S\left(\Delta K_{r},K_{x}\right)\;\approx \exp\left\{-j\left[\Phi_{0}\left(K_{x};x,r\right)+\Phi_{1}\left(K_{x};x,r\right)\cdot \Delta K_{r}+\Phi_{2}\left(K_{x};r\right)\cdot \Delta K_{r}^{2}\right]\right\}$$

where,

(8) $$\Phi_{0}\left(K_{x};x,r\right)=\left[\sqrt{K_{rc}^{2}-{\left(K_{x}+K_{rc}\sin\theta \right)}^{2}}\cos\theta +\left(K_{x}+K_{rc}\sin\theta \right)\sin\theta \right]\cdot r+\left(K_{x}+K_{rc}\sin\theta \right)\cdot x$$

(9) $$\Phi_{1}\left(K_{x};x,r\right)=\left[\frac{K_{rc}\cos^{2}\theta -K_{x}\sin\theta }{\sqrt{K_{rc}^{2}-{\left(K_{x}+K_{rc}\sin\theta \right)}^{2}}}\cos\theta +\sin^{2}\theta \right]\cdot r+x\sin\theta$$

(10) $$\Phi_{2}\left(K_{x};r\right)=-\frac{K_{x}^{2}}{2{\left[K_{rc}^{2}-{\left(K_{x}+K_{rc}\sin\theta \right)}^{2}\right]}^{\frac{3}{2}}}\cdot r\cos\theta +\frac{c^{2}}{16\pi \gamma }$$

It should be specified that $\Phi_{1}\left(K_{x};x,r\right)$ in (7) is the RCM after the RWC process. It is found that RCM is related to the azimuth position *x* and range *r* of a target, so RCM is both range- and azimuth-dependent. For CSA in the azimuth wavenumber domain, it is efficient to remove range-dependent RCM using a scaling process. However, the residual spatial-variant RCM caused by RWC still affects the azimuth focusing performance. The source of error will be discussed in the following subsection.

### 3.3. Coarse RCMC and Error Analysis

What followed is the coarse RCMC. In the CSA procedure, it is more convenient to compensate the range-variant RCM by a scaling process without range blocking. Transform (7) into the range time domain, so we have

(11) $$S\left(\hat{r},K_{x}\right)=\int S\left(\Delta K_{r},K_{x}\right)\cdot \exp\left(j\Delta K_{r}\hat{r}\right)d\Delta K_{r}$$

According to POSP, (11) is calculated by

(12) $$S\left(\hat{r},K_{x}\right)=\exp\left[-j\Phi_{0}\left(K_{x};x,r\right)\right]\cdot \exp\left\{j\frac{1}{4\Phi_{2}\left(K_{x};r\right)}{\left[\hat{r}-\Phi_{1}\left(K_{x};x,r\right)\right]}^{2}\right\}$$

In order to adjust the range-variant RCM, chirp scaling coefficient $a\left(K_{x}\right)$ is given by

(13) $$a\left(K_{x}\right)=\frac{K_{rc}\cos^{2}\theta -K_{x}\sin\theta }{\sqrt{K_{rc}^{2}-{\left(K_{x}+K_{rc}\sin\theta \right)}^{2}}}\cos\theta +\sin^{2}\theta -1$$

**Figure 3:**
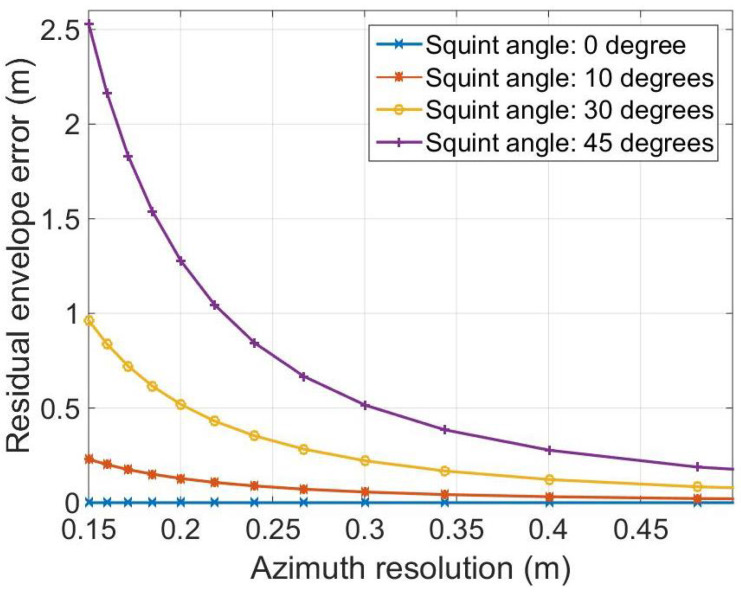
Residual envelope error of the target under 9 GHz.

So we have $\Phi_{1}\left(K_{x};x,r\right)=a\left(K_{x}\right)r+r+x\sin\theta$. Then, the chirp scaling function is designed to

(14) $$H_{cs}\left(\hat{r},K_{x}\right)=\exp\left\{j\frac{a\left(K_{x}\right)}{4\Phi_{2}\left(K_{x};r_{c}\right)}{\left[\hat{r}-a\left(K_{x}\right)r_{c}-r_{c}\right]}^{2}\right\}$$

where, $r_{c}$ represents the slant range of the scene center. Multiplying (12) by (14), and make

(15) $$\Delta r\left(K_{x},x\right)=-x\sin\theta \frac{a\left(K_{x}\right)}{a\left(K_{x}\right)+1}$$

represents the residual RCM. The numerical analysis of the residual RCM Δr in (15) is given in Figure 3, which gives the graphs of Δr as a function of azimuth resolution at a carrier frequency of 9 GHz. The target point simulated for the test is located at x=200 m. It is shown that the residual envelope error is obvious, especially for a high azimuth resolution.

The signal expression after scaling process is approximately given by

(16) $$\begin{array}{c} S_{cs}\left(\hat{r},K_{x}\right)=S\left(\hat{r},K_{x}\right)\cdot H_{cs}\left(\hat{r},K_{x}\right)\\ =\exp\left\{-j\left[\Phi_{0}\left(K_{x};x,r\right)+\Phi_{re1}\left(K_{x};r\right)+\Phi_{re2}\left(K_{x};x,r\right)\right]\right\}\\ \cdot \exp\left\{j\frac{a\left(K_{x}\right)+1}{4\Phi_{2}\left(K_{x};r\right)}{\left[\hat{r}-a\left(K_{x}\right)r_{c}-\left(r+x\sin\theta \right)-\Delta r\left(K_{x},x\right)\right]}^{2}\right\} \end{array}$$

where, the residual phases $\Phi_{re1}\left(K_{x};r\right)$ and $\Phi_{re2}\left(K_{x};r,x\right)$ are given by

(17) $$\Phi_{re1}\left(K_{x};r\right)=-\frac{1}{4\Phi_{2}\left(K_{x};r\right)}a\left(K_{x}\right)\left[a\left(K_{x}\right)+1\right]{\left(r-r_{c}\right)}^{2}$$

(18) Φre2Kx;x,r=−14Φ2Kx;r2aKxr−rcxsinθ+x2sin2θaKxaKxaKx+1aKx+1

It is shown in (16) that the RCM after the chirp scaling process is azimuth-variant. Transform (16) into two-dimensional wavenumber domain, we have

(19) $$S_{cs}\left(\Delta K_{r},K_{x}\right)=\int S_{cs}\left(\hat{r},K_{x}\right)exp\left(-j\Delta K_{r}\hat{r}\right)d\hat{r}$$

According to POSP, (19) is calculated by

(20) $$\begin{array}{c} S_{cs}\left(\Delta K_{r},K_{x}\right)=\exp\left\{-j\left[\Phi_{0}\left(K_{x};x,r\right)+\Phi_{re1}\left(K_{x};r\right)+\Phi_{re2}\left(K_{x};x,r\right)\right]\right\}\\ \cdot \exp\left[-j\frac{\Phi_{2}\left(K_{x};r\right)}{a\left(K_{x}\right)+1}\Delta {K_{r}}^{2}\right]\cdot exp\left\{-j\Delta K_{r}\left[a\left(K_{x}\right)r_{c}+\left(r+x\sin\theta \right)+\Delta r\left(K_{x},x\right)\right]\right\} \end{array}$$

It is shown in (20) that the third phase term is the quadratic phase term. The fourth phase term is the primary phase term, which represents the RCM of signal. The RCM is azimuth-variant due to the processing of RWC. Hence, the range compression and second range compression function are

(21) $$H_{mf}\left(K_{x};r_{c}\right)=\exp\left[j\frac{\Phi_{2}\left(K_{x};r_{c}\right)}{a\left(K_{x}\right)+1}\Delta {K_{r}}^{2}\right]$$

The RCMC function in CSA is

(22) $$H_{rcmc}\left(K_{x};r_{c}\right)=\exp\left[ja\left(K_{x}\right)r_{c}\Delta K_{r}\right]$$

Multiplying (20) with (21) and (22), we have

(23) $$\begin{array}{c} S_{cs}\left(\Delta K_{r},K_{x}\right)=\exp\left[-j\Phi_{0}\left(K_{x};x,r\right)\right]\cdot \exp\left[-j\Phi_{re1}\left(K_{x};r\right)\right]\\ \cdot \exp\left[-j\Phi_{re2}\left(K_{x};x,r\right)\right]\cdot exp\left\{-j\Delta K_{r}\left[\left(r+x\sin\theta \right)+\Delta r\left(K_{x},x\right)\right]\right\} \end{array}$$

Transform (23) into the range time domain by range IFT. Then, compensate the residual azimuth-invariant phase $\Phi_{re1}\left(K_{x};r\right)$, the signal is then given by

(24) $$\begin{array}{c} S_{cs}\left(\hat{r},K_{x}\right)=\mathrm{sinc}\left\{\frac{2\alpha T_{p}}{c}\left[\hat{r}-\left(r+x\sin\theta \right)-\Delta r\left(K_{x},x\right)\right]\right\}\\ \cdot \exp\left[-j\Phi_{0}\left(K_{x};x,r\right)\right]\cdot \exp\left[-j\Phi_{re2}\left(K_{x};x,r\right)\right] \end{array}$$

Transform (24) into the azimuth time domain by azimuth IFT, we have

(25) $$\begin{array}{c} s_{cs}\left(\hat{r},X\right)=\int S_{cs}\left(\hat{r},K_{x}\right)\exp\left(jK_{x}X\right)dK_{x}\\ =\mathrm{sinc}\left\{\frac{2\alpha T_{p}}{c}\left[\hat{r}-\left(r+x\sin\theta \right)-\Delta r\left(K_{x}^{*},x\right)\right]\right\}\\ \;\;\cdot \exp\left[-j\Phi_{0}\left(K_{x}^{*};x,r\right)\right]\cdot \exp\left[-j\Phi_{re2}\left(K_{x}^{*};x,r\right)\right] \end{array}$$

where, $K_{x}^{*}$ represents the stationary phase point. According to POSP, and ignoring the effect of the spatial-variant phase term $\Phi_{re2}$, the stationary phase point is approximately calculated by

(26) $$K_{x}^{*}\left(X;x,r\right)\approx -\frac{X-x-r\sin\theta }{\sqrt{{\left(r\cos\theta \right)}^{2}+{\left(X-x-r\sin\theta \right)}^{2}}}K_{rc}-K_{rc}\sin\theta$$

Substitute (26) into (25), then the explicit expressions of $\Phi_{0}\left(X;x,r\right)$ are given by

(27) $$\Phi_{0}\left(X;x,r\right)=K_{rc}\cdot \left[\sqrt{{\left(r\cos\theta \right)}^{2}+{\left(X-x-r\sin\theta \right)}^{2}}+X\sin\theta \right]$$

The residual RCM is then expressed in azimuth time domain by

(28) $$\Delta r\left(X;x,r\right)=-x\sin\theta \left[1-\frac{r}{\sqrt{{\left(r\cos\theta \right)}^{2}+{\left(X-x-r\sin\theta \right)}^{2}}+\left(X-x\right)\sin\theta }\right]$$

For high-resolution SAR imaging, the residual RCM $\Delta r\left(X;x,r\right)$ is non-negligible and its precise correction is necessary to yield optimal focus performance. The residual RCM is azimuth-dependent. This fact paves a way for us to correct the residual RCM with SA processing. In order to deal with the spatial dependence of $\Delta r\left(X;x,r\right)$, we propose a new approach to correct it in the SA-image domain.

## 4. Fine Rcmc for SA Processing

### 4.1. Residual Fine RCMC

In practical applications, the real-time processing capability is usually limited by the computing power and memory of the processing devices on the machine, which makes it impossible to calculate and store large amounts of data. For high-precision and highly squinted SAR imaging, the image post-filtering method [23] can achieve accurate RCMC, but this strategy is difficult to apply in real-time processing. Therefore, in order to facilitate a real-time processing process, the SA processing strategy should be adopted in the fine RCMC.

**Figure 4:**
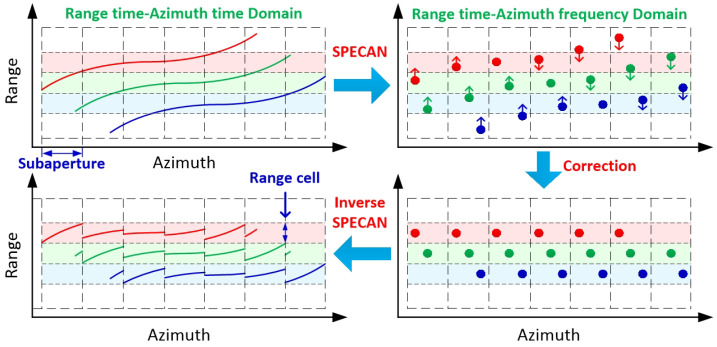
Schematic diagram of the fine RCMC processing based on SA processing.

The schematic diagram of the fine RCMC processing based on SA processing is given in Figure 4. It is more efficient to constrain the RCM in each SA into one range cell rather than absolutely correcting them. The reason for this is that it is unnecessary to correct RCM entirely but to a precise level to ensure an optimal azimuth compression, such as a half or a quarter of one range cell. The SPECAN algorithm is then available to focus the targets in each SA, which gives us a way to efficiently correct the azimuth-variant RCM by shifting the targets in each SA to the correct range cells. In the following content, we will introduce the proposed fine RCMC in the SA image domain in detail.

At first, we segment the data into a sequence of SA data sets. The azimuth coordinate of the gth SA is given by $X_{g}=U_{g}+X^{\prime }$, where X′∈−LsaLsa22,LsaLsa22, $U_{g}$ is the gth SA center and $L_{sa}$ is the SA length. SPECAN imaging is then introduced by a deramping process to obtain low-resolution images of each SA. The deramping process is implemented by multiplying (25) with the reference phase function $H_{Ref}\left(X_{g},r\right)$ in (29), which is given by

(29) $$H_{Ref}\left(X_{g},r\right)\;=\exp\left[j\Phi_{0}\left(X_{g};0,r\right)\right]$$

where it is noted in (29) that $H_{Ref}\left(X_{g},r\right)$ is constructed respect to the azimuth center at *r*. The deramped signal is shown as

(30) $$\begin{array}{c} S\left(\hat{r},X_{g}\right)\;=\mathrm{sinc}\left\{\frac{2\alpha T_{p}}{c}\left[\hat{r}-\left(r+x\sin\theta \right)-\Delta r\left(X_{g};x,r\right)\right]\right\}\\ \cdot \exp\left\{-j\left[\Phi_{0}\left(X_{g};x,r\right)-\Phi_{0}\left(X_{g};0,r\right)\right]\right\}\cdot \exp\left[-j\Phi_{re2}\left(X_{g};x,r\right)\right] \end{array}$$

In order to determine the mapping relationship between the target coordinate and pixels in the SA image, we ignore the inoperative spatial-variant phase and rewrite the invariant phase function in (30) as follows

(31) $$\begin{array}{c} \Delta \Phi_{0}\left(X_{g};x,r\right)=\Phi_{0}\left(X_{g};x,r\right)-\Phi_{0}\left(X_{g};0,r\right)\\ =\psi_{0}+H_{a1}\cdot X_{g}+H_{a2}\cdot X_{g}^{2}+O\left(X_{g}^{3}\right) \end{array}$$

where,

(32) $$\psi_{0}=K_{rc}\cdot \left[\sqrt{{\left(r\cos\theta \right)}^{2}+{\left(x+r\sin\theta \right)}^{2}}-r\right]$$

(33) $$H_{a1}=-K_{rc}\cos^{2}\theta \left[\frac{x}{r}-\frac{3}{2}\sin\theta \frac{x^{2}}{r^{2}}-\frac{1}{2}\left(1-5\sin^{2}\theta \right)\frac{x^{3}}{r^{3}}\right]$$

(34) $$H_{a2}=-K_{rc}\cos^{2}\theta \sin\theta \frac{x}{r^{2}}$$

By azimuth FT to (30), the signal is transformed into the azimuth wavenumber domain, and the low-resolution SPECAN image is obtained. The question arose as to how to calculate the real coordinate for each image bin in the SPECAN image plane. For a target with coordinate $\left(K_{a},r_{s}\right)$ in the SPECAN image, the mapping relationship with its coordinate $\left(x,r\right)$ is given by

(35) $$r_{s}=r+x\sin\theta$$

(36) $$K_{a}=-H_{a1}-2H_{a2}U_{g}\approx a_{1}x+a_{2}x^{2}+a_{3}x^{3}$$

where,

(37) $$a_{1}\approx K_{rc}\cos^{2}\theta \left(\frac{1}{r_{s}}+2U_{g}\frac{1}{r_{s}^{2}}\sin\theta \right)$$

(38) $$a_{2}\approx -\frac{3}{2}K_{rc}\cos^{2}\theta \sin\theta \frac{1}{r_{s}^{2}}$$

(39) $$a_{3}\approx -\frac{1}{2}K_{rc}\cos^{2}\theta \left(1-5\sin^{2}\theta \right)\frac{1}{r_{s}^{3}}$$

The MSR is used for expressing *x* as a function of $r_{s}$ and $K_{a}$, and the range coordinate *r* is found correspondingly. The mapping from each image bin to a real target coordinate is given by

(40) $$x=b_{1}\cdot K_{a}+b_{2}\cdot K_{a}^{2}+b_{3}\cdot K_{a}^{3}$$

(41) $$r=r_{s}-\sin\theta \left(b_{1}\cdot K_{a}+b_{2}\cdot K_{a}^{2}+b_{3}\cdot K_{a}^{3}\right)$$

where, $b_{1}=a_{1}^{-1}$, $b_{2}=-a_{1}^{-3}a_{2}$ and $b_{3}=a_{1}^{-5}\left(2a_{2}^{2}-a_{1}a_{3}\right)$. By using this mapping relationship between the spatial domain and azimuth wavenumber domain, we can calculate the residual RCM at $X=U_{g}$ by substituting (40) and (41) into $\Delta r\left(X;x,r\right)$ in (28), which is expressed as $\Delta r\left(X;K_{a},r\right)$. Then, we can correct the residual RCM $\Delta r\left(X;K_{a},r\right)$ in the SPECAN image domain by an interp processing, and this process can be represented as

(42) $$r\left(X;K_{a}\right)+\Delta r\left(X;K_{a},r\right)\to r\left(X;K_{a}\right)$$

The inversed SPECAN will be processed by an azimuth IFT in the SPECAN image domain and then by multiplying a phase term ${H^{*}}_{Ref}\left(X_{g},r\right)$, which is shown by

(43) $${H^{*}}_{Ref}\left(X_{g},r\right)=\exp\left[-j\Phi_{0}\left(X_{g};0,r\right)\right]$$

### 4.2. SA Length Analysis

In the sub-aperture RCMC approach, the data are segmented into several azimuth SA blocks, such as *G* SA blocks. The image-domain RCMC is performed in sequence for all SA block data before combining them together with the full-aperture data. The following question arises immediately: How to determine the SA length to ensure a high accuracy of residual RCMC in the sub-aperture image domain. It should be noted that, after the fine RCMC with $\Delta r\left(U_{g};x,r\right)$, the spatial variant part $\Delta r\left(X;x,r\right)-\Delta r\left(U_{g};x,r\right)$ is left, and it should be constrained within a small extent, such as a half (or a quarter) of one range cell. Despite the conspicuous fact that the shorter SA is, the easier it is to meet this requirement, the fine RCMC with short SA usually involves a large computational load for RCMC processing. Thus, to derive a maximum SA length for high efficiency, the first-order derivative of $\Delta r\left(X;x,r\right)$ with respect to *X* is given by

(44) $$\frac{\partial \Delta r\left(X;x,r\right)}{\partial X}\approx \frac{r_{s}\cos^{2}\theta }{r^{2}}\cdot \left(X-x\right)$$

To constrain the variance of the residual RCM within each SA to be nominal enough, the following condition should be satisfied.

(45) $$\max\left[\left|\frac{r_{s}\cos^{2}\theta }{r^{2}}\cdot \left(X-x\right)\right|\cdot L_{sa}\right]\leq \frac{\delta r}{2},\begin{array}{cc} & \left(X-x\right)\in \left[-2L,2L\right] \end{array}$$

where, max[] denotes the maximum of a function and δr denotes the size of a range cell. By simplification, the condition indicates a proper SA length satisfying that

(46) $$L_{sa}\leq \frac{r^{2}}{4r_{s}L\cos^{2}\theta }\delta r$$

In the SA segmentation, overlapping and truncation are required to avoid discontinuities after the fine RCMC in the full-aperture data.

### 4.3. Computational Burden Analysis

After the fine RCMC, the azimuth frequency rates of targets in the same range cell are equalized via the ANLCS algorithm. Then, the identical azimuth matched filter can be applied in the range cell to achieve accurate azimuth compression for all targets simultaneously. A negative aspect of the fine RCMC with SA processing results is the increasing computational load. In this subsection, we will theoretically discuss the computational load of the proposed algorithm. Assume the size of full-aperture data is M×N (range × azimuth), the number of azimuth block is $K_{N}$, and the overlapping ratio of the blocking is 11QQ. Hence, the size of each block is M×Q+1NQ+1NKNQKNQ. It is illustrated that a *N*-point FFT/IFFT operation contains $5N\log_{2}\left(N\right)$ floating-point operations (FLOPs) and a complex multiplication contains 6 FLOPs [32]. The additional FLOP number for the fine RCMC stage is given by

(47) $$\begin{array}{c} C_{b}=K_{N}\left[\begin{array}{c} 3\times 6\times \frac{\left(Q+1\right)MN}{QK_{N}}+2\times 5\times M\times \frac{\left(Q+1\right)N}{QK_{N}}\log_{2}\left(M\right)\\ +2\times 5\times M\times \frac{\left(Q+1\right)N}{QK_{N}}\log_{2}\left(\frac{\left(Q+1\right)N}{QK_{N}}\right) \end{array}\right]\\ \;\;\;\;=\frac{18\left(Q+1\right)MN}{Q}+\frac{10\left(Q+1\right)MN}{Q}\log_{2}\left(\frac{\left(Q+1\right)MN}{QK_{N}}\right) \end{array}$$

It is shown in (47) that increasing $K_{N}$ can reduce the computational burden of the algorithm to a certain extent. Although the computational load of accurate RCMC is relatively increased, fortunately, as the fine RCMC is independent for different azimuth blocks, parallel processing is an efficient way to perform each SA data processing.

## 5. Experiments

### 5.1. Simulated Data

**Figure 5:**
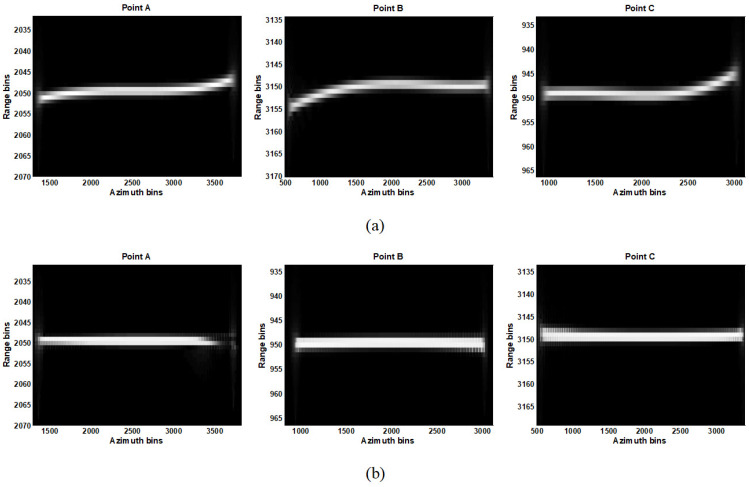
RCMC comparisons of chirp scaling algorithm (CSA) and proposal. (**a**) RCMC of points A, B, and C using CSA; (**b**) RCMC of A, B, and C using the proposed algorithm.

**Figure 6:**
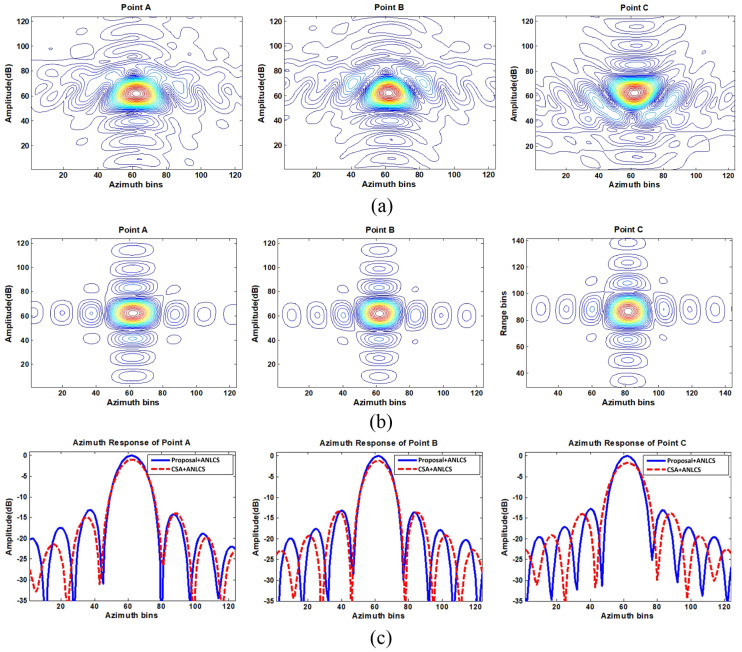
Azimuth impulse response comparisons. (**a**) 2D impulse responses of points A, B, and C using CSA + ANLCS; (**b**) 2D impulse response of A, B, and C using the proposed algorithm+ANLCS; (**c**) Comparison of azimuth impulse responses of points A, B, and C.

In this section, we validate the effectiveness of the proposed method with simulated strip-map SAR data. Table 1 lists the simulation parameters and three points, which consist of the relevant scene in the simulation. By using both CSA and the proposed fine RCMC approach, the range-compressed data in the azimuth time domain of points A, B and C are provided in Figure 5. Figure 5a gives the RCMC results with CSA and Figure 5b gives results with the proposed fine RCMC approach. By comparison, one can note the residual RCM after coarse RCMC is definitely not negligible for all points. The effectiveness of fine RCMC with the data segmentation processing is distinctive since the residual RCM is eliminated ideally as shown in Figure 5b. Then, ANLCS processing is implemented to the range-compressed data after RCMC to equalize the Doppler frequency rates. After, precise azimuth compression can be achieved with the identical matched filter. In order to show focus improvement by removing the residual spatial-variant RCM with the proposed data segmentation processing, 2D response impulses of different target points from the CSA + ANLCS and the Proposal + ANLCS are provided in Figure 6a,b, respectively. It is evident that the residual azimuth-variant RCM cannot be compensated by CSA + ANLCS, which causes significant smearing and energy spreading and leads to serious distortions of impulse response for all points. By contrast, the 2D impulse responses obtained by the Proposal + ANLCS are ideal. Sections of azimuth impulse response comparison of two RCMC methods are given in Figure 6c. Although the ANLCS processing provides highly accurate azimuth compression after RCMC with both CSA and the proposal, the focus degradation caused by the residual spatial-variant RCM in the CSA formation is significant enough to cause geometric and radiometric resolution losses. Focus evaluation with metrics is provided in Table 2. In order to present focus improvement to the fine RCMC, some evaluation metrics are utilized to evaluate the impulse responses of different target points. The evaluation metrics are peak side-lobe ratio (PSLR), integrated side-lobe ratio (ISLR), impulse response width (IRW) and peak value ratio (PVR). The PSLR defines the ratio of the peak value of the main lobe to the peak value of the strongest side lobe. The PSLR defines the ratio of the integrated value of the main lobe to the integrated value of ten side lobes nearby. The IRW defines the width of the main lobe at pulse amplitude drop 3dB. Smaller values of PSLR, ISLR and IRW mean a more accurate focus. The PVR defines the peak value ratio of the two main lobes used for comparison; a negative number represents the attenuation of the focusing energy. By removing the residual spatial-variant RCM via data segmentation processing, the resolution losses are very nominal and higher peak values are achieved, indicating significant focus improvement.

### 5.2. Real Measured Data

**Figure 7:**
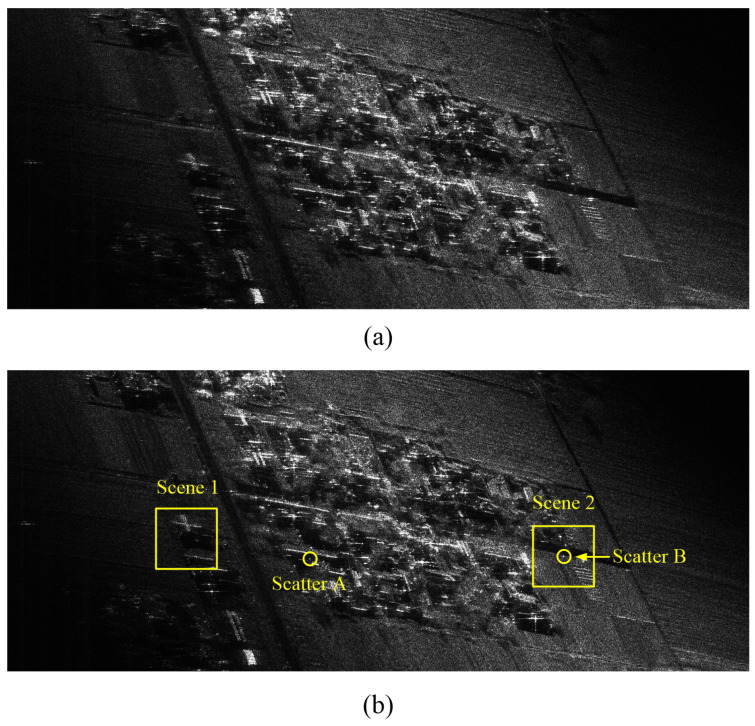
Scene imaging comparisons. (**a**) Processed by CSA + ANLCS; (**b**) Processed by Proposal + ANLCS.

**Figure 8:**
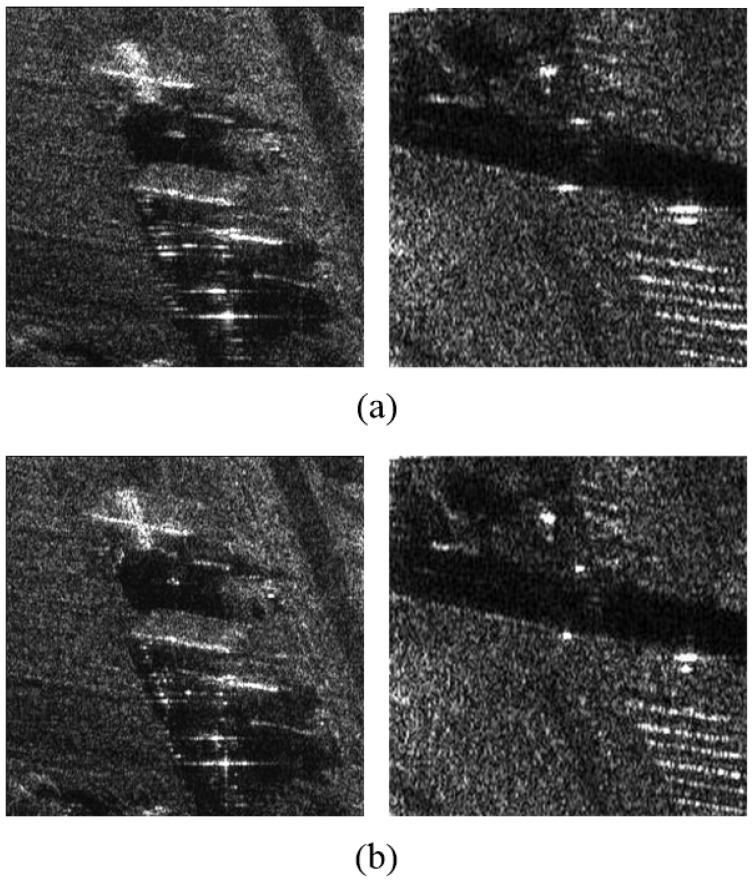
Local scene amplification imaging comparisons. (**a**) Processed by CSA + ANLCS; (**b**) Processed by Proposal + ANLCS.

In the following, we investigate whether the residual RCM issue is adequately solved by the proposed algorithm base on the raw high-resolution highly squinted airborne SAR data. The test data set was collected using an experimental SAR system working on strip-map mode; the system parameters are shown in Table 3. By imaging the original echo data, we get two component image results, shown in Figure 7, where Figure 7a is processed by CSA + ANLCS and Figure 7b is processed by Proposal + ANLCS. As presented in Figure 7, in the full-scene SAR image results, some sub-scenes with different targets are selected in order to perform a complete comparison. Since residual RCM is insufficient to cause defocus of the whole image, it is difficult to find obvious focusing performance improvement in the comparison of Figure 7. The results can be more clearly compared by local amplification. Scenes 1 and 2 in Figure 7b are magnified to the performance evaluation of the algorithms, which are shown in Figure 8. Figure 8a,b are processed by CSA + ANLCS and Proposal + ANLCS, respectively. By comparing Figure 8a,b, we can clearly find the differences in algorithm performance. Due to the limitation of CSA method, residual RCM will affect the focusing performance of image details in Figure 8a. As a contrast, Figure 8b improves on this problem, and the image details become clearer.

**Figure 9:**
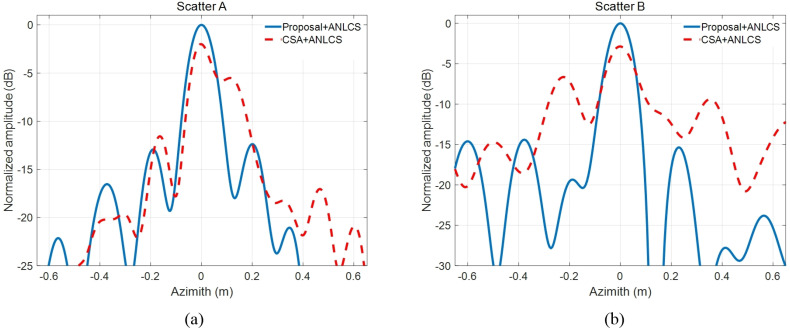
Azimuth impulse response comparisons. (**a**) Scatter A. (**b**) Scatter B.

Scatter A and Scatter B are strong scatters in the experimental scene. In order to compare the two methods more quantitatively, the azimuth pulse response functions of scatter A and scatter B processed by two contrastive RCMC methods are shown in Figure 9a,b, respectively. The quantitative analysis results of azimuth pulse response functions in Figure 9 are shown in Table 4. From the impulse response curve in Figure 9, we can see that the results obtained by the proposed method are closer to the ideal pulse curve. This conclusion can also be drawn from the quantitative analysis results in Table 4 because of the presence of smaller PSLR, ISLR and IRW and larger PVR of the proposed method. According to the comparisons above, we conclude that the proposed fine RCMC approach is effective and satisfactory to focus the high-resolution highly squinted SAR imagery. This result confirms the effectiveness of the proposed algorithm.

### 5.3. Discussion

Through the analysis of simulation and measured data, we can carry out further discussion. In the quantitative analysis of indicators, PVR is defined as the ratio of the peak value of response from the CSA + ANLCS in azimuth and that of the proposed method. Due to the fact that the residual RCM causes blurs and extensions of impulse response, the peak value from the CSA + ANLCS is deemed to be smaller than that from the proposal. The target profile is constrained within a single range cell after fine RCMC. Consequently, the PVR turns out smaller than unity. Small peak value mean incomplete coherent integral, yielding a low signal-to-noise ratio together with geometric and radiometric resolution losses in the SAR image. In real measured data images, due to the existence of residual RCM, the image has some blurring, leading to significant resolution loss, while images obtained by the proposed algorithm are well-focused over the whole scene. It should be emphasized that, as we perform identical motion compensation with the position measurements, the focus superiority of the image from the proposed method over the image from conventional method is illustrated by the fine RCMC with the SA processing.

## 6. Conclusions

In this paper, we investigated a precise RCMC method for highly squinted SAR imagery. The main contribution is introducing an SA processing technique to correct the residual spatial-variant RCM in the SPECAN image domain. Based on rigid derivation of the analytical expression of spatial-variant residual RCM, the fine RCMC was implemented by image shift in the SPECAN image domain. Together with the azimuth frequency rates equalization with the ANLCS processing, the proposed method provides an ideal focus performance for highly squinted SAR imagery with high resolution. The selection of data segmentation length in range and azimuth dimensions as well as the computational burdens are also investigated in detail. Both simulated and real measured SAR data sets are experimented to validate the effectiveness of the proposed method.

## Figures and Tables

**Table 1:** Simulation parameters.

Parameter	Value
Working band	X-band
Center closest slant range	1 km
Squint angle	45 degrees
Range resolution	0.15 m
Azimuth resolution	0.15 m
Point A coordinate	(1000, 75)
Point B coordinate	(1200, 0)
Point C coordinate	(800, 0)

**Table 2:** Focus performance comparsions.

Target Point	Approach	PSLR (dB)	ISLR (dB)	IRW (m)	PVR (dB)
A	CSA + ANLCS	−13.03	−9.43	0.22	−0.94
A	Proposal + ANLCS	−13.14	−10.14	0.16	0
B	CSA + ANLCS	−13.10	−9.31	0.191	−1.14
B	Proposal + ANLCS	−13.35	−10.03	0.16	0
C	CSA + ANLCS	−12.40	−10.10	0.217	−1.94
C	Proposal + ANLCS	−13.03	−11.40	0.16	0

**Table 3:** System parameters.

Parameter	Value
Working band	Ka-band
Center closest slant range	4 km
Velocity	70 m/s
Squint angle	40 degrees
Range resolution	0.2 m
Azimuth resolution	0.2 m

**Table 4:** Focus performance comparsions.

Scatter Point	Approach	PSLR (dB)	ISLR (dB)	IRW (m)	PVR (dB)
A	CSA + ANLCS	−3.05	−2.48	0.30	−2.00
A	Proposal + ANLCS	−10.75	−8.82	0.18	0
B	CSA + ANLCS	−3.28	−2.04	0.23	−2.87
B	Proposal + ANLCS	−13.33	−12.87	0.16	0

## Data Availability

Real measured data is provided by Beijing Institute of Radio Measurement.
